# Apolipoprotein E Induced Cognitive Dysfunction: Mediation Analysis of Lipids and Glucose Biomarkers in an Elderly Cohort Study

**DOI:** 10.3389/fnagi.2021.727289

**Published:** 2021-08-13

**Authors:** Linxin Liu, Huichu Li, Hari Iyer, Andy J. Liu, Yi Zeng, John S. Ji

**Affiliations:** ^1^Environmental Research Center, Duke Kunshan University, Suzhou, China; ^2^Harvard TH Chan School of Public Health, Harvard University, Boston, MA, United States; ^3^Division of Population Sciences, Dana-Farber Cancer Institute, Boston, MA, United States; ^4^Duke Department of Neurology, Duke University School of Medicine, Durham, NC, United States; ^5^Center for Healthy Aging and Development Studies, National School of Development, Peking University, Beijing, China; ^6^Duke Center for the Study of Aging and Human Development, Duke Medical School, Durham, NC, United States; ^7^Vanke School of Public Health, Tsinghua University, Beijing, China; ^8^Global Health Research Center, Duke Kunshan University, Suzhou, China

**Keywords:** *APOE*, lipids, glucose, cognitive function, mediation analysis

## Abstract

**Introduction:**

Prior evidence suggested *Apolipoprotein E (APOE)*, lipids, and glucose metabolism may act through the same pathways on the pathogenesis of Alzheimer’s disease (AD).

**Methods:**

This prospective study used data from the Chinese Longitudinal Healthy Longevity Study. We tested the associations of *APOE* genotype (ε2ε2, ε2ε3, ε2ε4, ε3ε3, ε3ε4, and ε4ε4) and cognitive function using generalized estimating equations (GEE). We examined for possible mediation and effect modification by lipids and glucose level in this association.

**Results:**

*APOE* ε*2* showed significant direct protective effect and indirect harmful effect through TC on cognitive function. Abnormal lipids or glucose levels were not consistently associated with cognitive dysfunction in our study. We did not detect significant indirect effects through lipids for *APOE* ε*4* or any indirect effects through glucose.

**Discussion:**

These findings suggested complicated relationships among *APOE*, lipids, glucose, and cognitive function. Further study can make validations in other populations.

## Introduction

*Apolipoprotein E (APOE)* gene is a well-established susceptibility gene for the development of late-onset Alzheimer’s disease (AD). Apolipoprotein E (ApoE) protein belongs to a family of fat-binding proteins responsible for transporting cholesterol. The *APOE* gene could affect neurodegeneration through multiple pathways, including neurite remodeling, glutamate receptor function, and synaptic plasticity modulation, and cholesterol redistribution (Huang and Mucke, 2012).

There are three common alleles (ε2, ε3, and ε4) in the *APOE* gene, resulting in 6 *APOE* genotypes. A meta-analysis of 141 articles covering European, North American, and East Asian population demonstrated that *APOE* genotype had a positive association with total cholesterol (TC) and low-density lipoprotein cholesterol (LDL-C) levels when ordered as ε2/ε2, ε2/ε3, ε2/ε4, ε3/ε3, ε3/ε4 and ε4/ε4 (Bennet et al., 2007). It is also well established that lipids are crucial in the development and functioning of the central nervous system (CNS; de Chaves and Narayanaswami, 2008). A growing body of evidence suggests that glucose hypometabolism may be a key player in dementia pathology (Kuehn, 2019). The effects of *APOE* polymorphism may also act through some of the same mechanisms as the disruption of homeostasis in lipid and glucose metabolism in the pathogenesis of AD (Shinohara and Sato, 2019).

Most studies have focused on the interactions between *APOE* polymorphisms and cholesterol on cognitive function, while few reported the possible mediation by cholesterol or glucose for *APOE* on cognitive function. Some studies reported that increasing cholesterol was associated with AD risk only in ε4 non-carriers, not in ε4 carriers (Evans et al., 2000; Hall et al., 2006). In a Chinese population, low serum high-density lipoprotein cholesterol (HDL-C) levels had a higher risk for cognitive dysfunction only in ε4 carriers, while different types of cholesterol (TC, HDL-C, LDL-C, and triglycerides) were not associated with cognitive function in ε4 non-carriers (Wei et al., 2020). In contrast, some studies reported the effect of cholesterol on cognitive function was independent of *APOE* (Toro et al., 2014; An et al., 2019), and the high-cholesterol subgroup had a higher cognitive decline rate than the normal-cholesterol subgroups with or without *APOE* ε4 (Evans et al., 2004). Despite the inconsistent findings, we aim to investigate the interaction and mediation among *APOE*, cholesterol, glucose level, and how it impacts cognition using a large cohort of elderly Chinese participants.

## Materials and Methods

### Study Population

We used data from the 2012 wave of the Chinese Longitudinal Healthy Longevity Study (CLHLS). The study collected blood samples for biomarker examinations in eight longevity areas (Laizhou City of Shandong Province, Xiayi County of Henan Province, Zhongxiang City of Hubei Province, Mayang County of Hunan Province, Yongfu County of Guangxi Autonomous Area, Sanshui District of Guangdong Province, Chengmai County of Hainan Province and Rudong County of Jiangsu Province). The published cohort profile described the study design and sample method (Lv et al., 2019). A part of samples received genetic sequencing. Two thousand and thirty hundred thirty two participants had the blood sample collected in 2012. Participants who did not have genetic data (*n* = 321), cholesterol data (*n* = 42), essential covariates data (*n* = 279), or younger than 65 (*n* = 63) were excluded. After exclusions, there were 1627 (70%) participants with 3379 MMSE measurements in the study (shown in Supplementary Figure 1).

### *APOE* Genotyping

The Beijing Genomics Institute (BGI) performed the genomic sequencing using a customized chip based on the previous CLHLS Genome-Wide Association Study (GWAS). Genotyping and quality control procedures were reported previously (Zeng et al., 2016). The chip targeted about 27 k longevity-phenotype related single nucleotide polymorphisms (SNPs). We used rs429358 and rs7412 to code the three common allelic variants of *APOE*: *APOE2* (rs429358: rs7412 = T:T), *APOE3* (rs429358: rs7412 = T:C), and *APOE4* (rs429358: rs7412 = C:C) and classified our participants into 6 *APOE* genotypes (ε2ε2, ε2ε3, ε2ε4, ε3ε3, ε3ε4, and ε4ε4). We then categorized the genotypes into ε2 carriers (ε2ε2, ε2ε3), ε2ε4 carriers, ε3ε3 carriers, and ε4 carriers (ε3ε4, ε4ε4).

### Biomarker Measurement

The participants provided the blood sample at the same time as the baseline interview. The technician tested blood plasma biomarkers included fasting blood glucose (FBG), glycated serum protein (GSP), total cholesterol (TC), triglyceride (TG), and high-density lipoprotein cholesterol (HDL-C) using an Automatic Biochemistry Analyzer (Hitachi 7180, Japan) with commercially available diagnostic kits (Roche Diagnostic, Mannheim, Germany) at Capital Medical University in Beijing. Low-density lipoprotein cholesterol (LDL-C) was calculated using the formula of Friedewald et al: LDL-C = TC-(HDL-C)-TG/5 (Friedewald et al., 1972). We also calculated the TC/HDL-C ratio and LDL-C/HDL-C ratio. We defined TC ≥ 3.8 mmol/L as high TC, TG ≥ 1.7 mmol/L as high TG, HDL-C < 1 mmol/L as low HDL-C, and LDL-C ≥ 2.6 mmol/L as high LDL-C based on the Chinese and American guidelines for cholesterol control (Joint Committee on Revision of Guidelines for Prevention and Treatment of Dyslipidemia in China, 2016; Grundy et al., 2019). We classified FBG (mmol/L) into four groups based on the Guidelines for the prevention and control of type 2 diabetes in China (2017 Edition): FBG < 3.9 as hypoglycemia, 6.1 > FBG ≥ 3.9 as normal blood glucose, 7 > FBG ≥ 6.1 as high blood glucose, and FBG ≥ 7 as diabetes (Chinese Diabetes Society, 2018).

### Cognitive Function

We used an adapted Chinese version of the Mini-Mental State Examination (MMSE) to assess the cognitive function of the participants. They took the first exam at the same time as the blood sample collection in the 2012 interview, and the follow-up examinations were given in 2014 and 2017/2018. The scale is 0–30 points, a higher score indicating better cognitive function. We defined cognitive dysfunction as MMSE < 24 scores.

### Covariates

We categorized the ethnicity as Han Chinese or other ethnic minorities in China. We used years in schools as a measure of literacy level. We classified marital status into two categories: currently married and living with the spouse, or not married (widowed/separated/divorced/never married/married but not living with the spouse). We classified city and town as “Urban” and village as “Rural.” We firstly divided the regular exercise, smoking, and alcohol drinking status into three categories: “Current,” “Former,” and “Never.” For example, participants were asked, “do you do exercise regularly at present (planned exercise like walking, playing balls, running and so on)?” and/or “did you do exercise regularly in the past?” We defined the regular exercise status as “Current” for participants who answered “Yes” to the first question, “Former” for who answered “No” to the first question and “Yes” to the second question, and “Never” for who answered “No” to both two questions. Then we further quantified the current smoker based on the number of times smoke (or smoked) per day:<20 times/day and ≥ 20 times/day. We also quantified the current alcohol drinker based on the kind of alcohol and how much drank per day. The unit of alcohol was a Chinese unit of weight called ‘Liang’ (50 gram). The level of alcohol consumption was calculated as drinks of alcohol per day, based on the beverage type and amount, assuming the following alcohol content by volume (v/v) typically seen in China: strong liquor 53%, weak liquor 38%, grape wine 12%, rice wine 15%, and beer 4% (Millwood et al., 2013). A standard drink was equal to 14.0 grams of pure alcohol according to the criterion of the Center for Disease Control and Prevention in the United States and moderate drinking is up to 1 drink per day for women and up to 2 drinks per day for men according to Dietary Guidelines for Americans 2015–2020. Therefore, we defined those drank equal or less than 14 g pure alcohol per day for the female or 28 g per day for the male as light drinker, otherwise heavy drinker. We calculated BMI as body weight divided by the square of the body height (unit: kg/m^2^). We used the WHO standard of BMI, which defined a BMI of < 18.5 kg/m^2^ as underweight, a BMI of 18.5 to 25 kg/m^2^ as normal weight, a BMI of ≥ 25 kg/m^2^ as overweight/obese. We defined hypertension as systolic blood pressure ≥ 140 mmHg and/or diastolic blood pressure ≥ 90 mmHg, and used the self-reported hospital diagnosed hypertension if the blood pressure measurement was missing.

### Statistical Analysis

**FIGURE 1:**
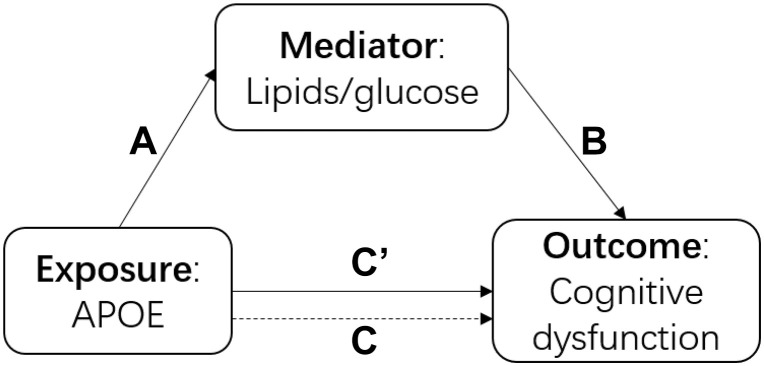
The diagram of the causal mediation analysis of *APOE* on cognitive function. A indicated the association between *APOE* and cholesterol; B indicated the association between lipids/glucose and cognitive function; C’ indicated the association between *APOE* and cognitive function after adjusted for lipids/glucose; C indicated the association between *APOE* and cognitive function without adjustment of lipids/glucose.

There were three main analyses in this study. First, we built a series of models to understand the association among the *APOE* polymorphism, blood cholesterol and cognitive function intuitively as shown in Figure 1. We performed a linear regression to examine the association between *APOE* genotype and each cholesterol and glucose biomarker (TC, TG, HDL-C, LDL-C, FBG, and GSP) separately (arrow A in Figure 1). We fitted generalized estimating equations (GEE) to test the association between each biomarker and cognitive function (measured as the continuous MMSE scores and cognitive dysfunction) separately (arrow B in Figure 1). We performed GEE of cognitive function on *APOE* genotype with and without adjustment for each biomarker (adjusted for the mediator: arrow C’ in Figure 1, not-adjusted for the mediator: arrow C in Figure 1). Secondly, we estimated the direct effect and indirect effect through cholesterol/glucose of *APOE* on the cognitive function using the causal mediation analysis (Vanderweele and Vansteelandt, 2009) based on the linear model using the genotype as the exposure, 2012 baseline biomarker as the mediator, and the mean MMSE score of 2012, 2014 and 2018 as the outcome. Thirdly, to examine the effect modification of biomarker on the association between *APOE* genotype and cognitive function, we added a product term of *APOE* genotype and the biomarker to test for interactions and used stratified analysis to estimate if the cholesterol or glucose levels modified the effect of *APOE*. We considered the same covariates for all the above models, adjusted for age, sex, ethnicity, and education firstly, then additionally adjusted for residence, marriage, exercise, smoking, and drinking alcohol, lastly further adjusted for BMI and hypertension. We used R 4.0.0 to perform all the analyses. We presented the 95% confidence interval for all the estimates.

## Results

### Population Characteristics

At baseline, we included participants aged from 65 to 110 years old, with a mean age at 84.8 (SD: 12.1). There are more females than males (52.1% vs. 47.9%). Han participants comprised the majority (92.7%). Education was low, at only 2.16 years on average (Median: 0), due to the historical circumstance at the time. The number of participants with cognitive dysfunction at baseline was 479 (29.4%), among which the mean age was 94.7 (SD: 8.2), and most were females (74.3%). *APOE* ε3ε3 was the most common genotype (68.7%). There were 291 (17.9%) ε2 carriers (ε2ε2, ε2ε3, ε2ε4), among which only 18 participants were ε2 homozygotes (ε2ε2) and 88 (30.2%) participants had cognitive dysfunction at baseline. There were 236 (14.5%) ε4 carriers (ε4ε2, ε4ε3, ε4ε4), among which only three participants were ε4 homozygotes (ε4ε4) and 79 (33.5%) participants had cognitive dysfunction at baseline. Distributions of age, gender, ethnicity, and education year were similar between ε2 carriers and ε2 non-carriers, ε4 carriers, and ε4 non-carriers. The mean (SD) of TC, TG, HDL-C, LDL-C, TC/HDL-C, LDL-C/HDL-C, FBG, GSP, and BMI were 4.30 (1.00) mmol/L, 0.989 (0.655) mmol/L, 1.29 (0.360) mmol/L, 2.56 (0.839) mmol/L, 3.53 (1.18), 2.12 (0.88), 4.66 (2.23) mmol/L, 240 (46.0) μmol/L, and 21.3 (4.46) kg/m^2^, respectively. The hypertension prevalence was 56.3%. Women had a higher mean age (88 vs. 80), higher baseline cognitive dysfunction prevalence (42% vs. 15.8%), and higher mean cholesterol level (TC: 4.48 vs. 4.11) compared to men (Table 1).

**TABLE 1:** Population baseline characteristics.

**Variables**	**Overall (*N* = 1627)**	**Baseline cognitive dysfunction**		**Gender**	
		**No (*N* = 1148)**	**Yes (*N* = 479)**	**Male (*N* = 779)**	**Female (*N* = 848)**
**MMSE score: Median (P25-P75)**	28 (21.5, 29)	29 (27, 30)	13 (3, 20)	29 (26, 30)	26 (15.75, 29)
**Cognitive dysfunction (Yes): *n* (%)**	479 (29.4)	/	/	123 (15.8)	356 (42.0)
**APOE Genotype: *n* (%)**					
ε2ε2	18 (1.1)	11 (1.0)	7 (1.5)	9 (1.2)	9 (1.1)
ε2ε3	255 (15.7)	183 (15.9)	72 (15.0)	119 (15.3)	136 (16.0)
ε2ε4	18 (1.1)	9 (0.8)	9 (1.9)	9 (1.2)	9 (1.1)
ε3ε3	1118 (68.7)	797 (69.4)	321 (67.0)	537 (68.9)	581 (68.5)
ε3ε4	215 (13.2)	146 (12.7)	69 (14.4)	103 (13.2)	112 (13.2)
ε4ε4	3 (0.2)	2 (0.2)	1 (0.2)	2 (0.3)	1 (0.1)
**TC (mmol/L): Mean (SD)**	4.30 (1.00)	4.37 (0.984)	4.14 (1.02)	4.11 (0.895)	4.48 (1.06)
**TG (mmol/L): Median (P25-P75)**	0.82 (0.59, 1.15)	0.845 (0.6, 1.22)	0.78 (0.57, 1.055)	0.76 (0.55, 1.075)	0.88 (0.66, 1.23)
**HDL-C (mmol/L): Mean (SD)**	1.29 (0.360)	1.30 (0.371)	1.26 (0.328)	1.26 (0.349)	1.32 (0.367)
**LDL-C (mmol/L): Mean (SD)**	2.56 (0.839)	2.60 (0.831)	2.48 (0.852)	2.43 (0.778)	2.68 (0.875)
**TC/HDL-C: Mean (SD)**	3.53 (1.18)	3.57 (1.23)	3.44 (1.03)	3.46 (1.09)	3.60 (1.25)
**LDL-C/HDL-C: Mean (SD)**	2.12 (0.880)	2.14 (0.885)	2.08 (0.867)	2.08 (0.876)	2.16 (0.883)
**FBG (mmol/L): Median (P25-P75)**	4.42 (3.67, 5.15)	4.4 (3.61, 5.10)	4.49 (3.82, 5.25)	4.44 (3.62, 5.155)	4.42 (3.71, 5.14)
**GSP (μmol/L): Mean (SD)**	240 (46.0)	243 (49.3)	231 (35.5)	240 (46.2)	239 (45.9)
**Female: *n* (%)**	848 (52.1)	492 (42.9)	356 (74.3)	0 (0)	848 (100)
**Age: Mean (SD)**	84.8 (12.1)	80.7 (11.0)	94.7 (8.21)	80.7 (10.8)	88.6 (12.0)
**Age group: *n* (%)**					
65∼	602 (37.0)	580 (50.5)	22 (4.6)	393 (50.4)	209 (24.6)
80∼	412 (25.3)	311 (27.1)	101 (21.1)	208 (26.7)	204 (24.1)
90∼	305 (18.7)	153 (13.3)	152 (31.7)	115 (14.8)	190 (22.4)
≥100	308 (18.9)	104 (9.1)	204 (42.6)	63 (8.1)	245 (28.9)
**Education year: Median (P25-P75)**	0 (0, 4)	1 (0, 5)	0 (0, 0)	3 (0, 6)	0 (0, 0)
**Education: *n* (%)**					
No formal education	977 (60.0)	563 (49.0)	414 (86.4)	263 (33.8)	714 (84.2)
1–6 years education	495 (30.4)	435 (37.9)	60 (12.5)	384 (49.3)	111 (13.1)
>6 years education	155 (9.5)	150 (13.1)	5 (1.0)	132 (16.9)	23 (2.7)
**Han: *n* (%) (vs. minorities)**	1509 (92.7)	1059 (92.2)	450 (93.9)	726 (93.2)	783 (92.3)
**Urban: *n* (%) (vs. rural)**	269 (16.5)	197 (17.2)	72 (15.0)	120 (15.4)	149 (17.6)
**Currently married: *n* (%) (vs. not married)**	665 (40.9)	597 (52.0)	68 (14.2)	478 (61.4)	187 (22.1)
**Exercise: n (%)**					
Never	1293 (79.5)	876 (76.3)	417 (87.1)	605 (77.7)	688 (81.1)
Former	52 (3.2)	34 (3.0)	18 (3.8)	23 (3.0)	29 (3.4)
Current	282 (17.3)	238 (20.7)	44 (9.2)	151 (19.4)	131 (15.4)
**Smoking: *n* (%)**					
Never	1202 (73.9)	790 (68.8)	412 (86.0)	409 (52.5)	793 (93.5)
Former	149 (9.2)	122 (10.6)	27 (5.6)	125 (16.0)	24 (2.8)
<20 times/day	153 (9.4)	118 (10.3)	35 (7.3)	127 (16.3)	26 (3.1)
≥20 times/day	123 (7.6)	118 (10.3)	5 (1.0)	118 (15.1)	5 (0.6)
**Alcohol drinking: *n* (%)**					
Never	1255 (77.1)	845 (73.6)	410 (85.6)	481 (61.7)	774 (91.3)
Former	97 (6.0)	80 (7.0)	17 (3.5)	76 (9.8)	21 (2.5)
≤14 (female) 28 (male) g/d	97 (6.0)	75 (6.5)	22 (4.6)	81 (10.4)	16 (1.9)
>14 (female) 28 (male) g/d	178 (10.9)	148 (12.9)	30 (6.3)	141 (18.1)	37 (4.4)
**BMI (kg/m2): Mean (SD)**	21.3 (4.46)	21.8 (4.24)	20.3 (4.79)	21.9 (4.18)	20.8 (4.63)
**Hypertension (Yes): *n* (%)**	916 (56.3)	642 (55.9)	274 (57.2)	405 (52.0)	511 (60.3)

### The Association Between *APOE* and Lipids/Glucose Level

According to the fully adjusted linear regression model of *APOE* genotypes and lipids/glucose level (Table 2), ε2 carriers had lower levels of TC (mmol/L), LDL-C (mmol/L), TC/HDL-C, and LDL-C/HDL-C ratio [Mean difference (95% confidence interval CI): −0.418 (−0.542, −0.295), −0.476 (−0.578, −0.374), −0.442 (−0.587, −0.297), and −0.442 (−0.55, −0.334) respectively], and higher HDL-C (mmol/L) [Mean difference (95% CI): 0.040 (−0.006, 0.086)] compared to ε3ε3 carriers. On the contrary, compared with the same reference group ε3ε3 carrier, ε4 carriers had higher levels of TC, LDL-C, TC/HDL-C, and LDL-C/HDL-C [Mean difference (95% CI): 0.083 (−0.052, 0.219), 0.119 (0.007, 0.231), 0.254 (0.095, 0.413), and 0.176 (0.058, 0.295) respectively], and lower HDL-C [Mean difference (95% CI): −0.055 (−0.105, −0.004)]. Increasing age was negatively associated with cholesterol [Mean difference (95% CI): −0.012 (−0.017, −0.007) for TC, −0.01 (−0.013, −0.007) for TG, −0.004 (−0.005, −0.002) for HDL-C, −0.004 (−0.008, 0.0002) for LDL-C] and not significantly associated with FBG or GSP. Female had higher cholesterol level than the male [Mean difference (95% CI): 0.565 (0.448, 0.683) for TC, 0.232 (0.154, 0.309) for TG, 0.079 (0.035, 0.123) for HDL-C, 0.384 (0.286, 0.482) for LDL-C] and there was no significant difference of FBG or GSP between them. We did not find significant interaction between *APOE* and age/sex on the cholesterol level. FBG, BMI, and hypertension were found not associated with *APOE*.

**TABLE 2:** Differences of lipids and glucose level by *APOE* genotype.

**Biomarker**	**Genotype**	***n***	**Change in biomarker (95% CI)^*a*^**	***p-*Value**	**Change in biomarker (95% CI)^*b*^**	***p*-Value**	**Change in biomarker (95% CI)^*c*^**	***p*-Value**
TC	ε3ε3 carrier	1118	Reference	/	Reference	/	Reference	/
TC	ε2 carrier	273	−0.431 (−0.555, −0.306)	<0.001	−0.427 (−0.551, −0.303)	<0.001	−0.418 (−0.542, −0.295)	<0.001
TC	ε2ε4 carrier	18	−0.254 (−0.691, 0.183)	0.255	−0.287 (−0.725, 0.151)	0.198	−0.335 (−0.772, 0.101)	0.132
TC	ε4 carrier	218	0.072 (−0.064, 0.208)	0.301	0.070 (−0.066, 0.205)	0.314	0.083 (−0.052, 0.219)	0.227
TG	ε3ε3 carrier	1118	Reference	/	Reference	/	Reference	/
TG	ε2 carrier	273	0.041 (−0.043, 0.124)	0.339	0.032 (−0.051, 0.115)	0.454	0.042 (−0.039, 0.122)	0.308
TG	ε2ε4 carrier	18	0.073 (−0.221, 0.367)	0.626	0.031 (−0.262, 0.325)	0.835	−0.016 (−0.301, 0.269)	0.911
TG	ε4 carrier	218	0.037 (−0.054, 0.129)	0.428	0.028 (−0.062, 0.119)	0.540	0.038 (−0.050, 0.126)	0.399
HDL-C	ε3ε3 carrier	1118	Reference	/	Reference	/	Reference	/
HDL-C	ε2 carrier	273	0.041 (−0.006, 0.088)	0.090	0.039 (−0.008, 0.086)	0.102	0.040 (−0.006, 0.086)	0.090
HDL-C	ε2ε4 carrier	18	0.109 (−0.057, 0.276)	0.198	0.073 (−0.092, 0.239)	0.385	0.093 (−0.069, 0.256)	0.261
HDL-C	ε4 carrier	218	−0.054 (−0.106, −0.002)	0.040	−0.051 (−0.102, 0)	0.051	−0.055 (−0.105, −0.004)	0.033
LDL-C	ε3ε3 carrier	1118	Reference	/	Reference	/	Reference	/
LDL-C	ε2 carrier	273	−0.489 (−0.593, −0.385)	<0.001	−0.479 (−0.582, −0.376)	<0.001	−0.476 (−0.578, −0.374)	<0.001
LDL-C	ε2ε4 carrier	18	−0.395 (−0.761, −0.029)	0.034	−0.375 (−0.739, −0.010)	0.044	−0.422 (−0.784, −0.060)	0.022
LDL-C	ε4 carrier	218	0.107 (−0.007, 0.221)	0.065	0.106 (−0.007, 0.219)	0.066	0.119 (0.007, 0.231)	0.038
TC/HDL-C	ε3ε3 carrier	1118	Reference	/	Reference	/	Reference	/
TC/HDL-C	ε2 carrier	273	−0.453 (−0.605, −0.301)	<0.001	−0.447 (−0.597, −0.296)	<0.001	−0.442 (−0.587, −0.297)	<0.001
TC/HDL-C	ε2ε4 carrier	18	−0.370 (−0.906, 0.165)	0.175	−0.327 (−0.860, 0.205)	0.228	−0.427 (−0.940, 0.085)	0.102
TC/HDL-C	ε4 carrier	218	0.245 (0.079, 0.412)	0.004	0.233 (0.068, 0.398)	0.006	0.254 (0.095, 0.413)	0.002
LDL-C/HDL-C	ε3ε3 carrier	1118	Reference	/	Reference	/	Reference	/
LDL-C/HDL-C	ε2 carrier	273	−0.452 (−0.565, −0.34)	<0.001	−0.442 (−0.553, −0.331)	<0.001	−0.442 (−0.55, −0.334)	<0.001
LDL-C/HDL-C	ε2ε4 carrier	18	−0.397 (−0.794, 0)	0.05	−0.343 (−0.736, 0.050)	0.087	−0.411 (−0.793, −0.030)	0.035
LDL-C/HDL-C	ε4 carrier	218	0.166 (0.043, 0.290)	0.008	0.161 (0.039, 0.283)	0.010	0.176 (0.058, 0.295)	0.003
FBG	ε3ε3 carrier	1118	Reference	/	Reference	/	Reference	/
FBG	ε2 carrier	273	−0.256 (−0.551, 0.038)	0.088	−0.227 (−0.521, 0.066)	0.129	−0.193 (−0.489, 0.103)	0.202
FBG	ε2ε4 carrier	18	−0.041 (−1.077, 0.994)	0.938	0.125 (−0.912, 1.163)	0.813	0.124 (−0.909, 1.156)	0.814
FBG	ε4 carrier	218	0.028 (−0.294, 0.351)	0.863	0.030 (−0.291, 0.352)	0.853	0.026 (−0.294, 0.346)	0.874
GSP	ε3ε3 carrier	1118	Reference	/	Reference	/	Reference	/
GSP	ε2 carrier	273	−4.088 (−10.175, 2.000)	0.188	−4.421 (−10.468, 1.625)	0.152	0.225 (−5.758, 6.207)	0.941
GSP	ε2ε4 carrier	18	−15.403 (−36.807, 6.002)	0.158	−13.623 (−35.006, 7.760)	0.212	−10.718 (−31.588, 10.151)	0.314
GSP	ε4 carrier	218	−1.704 (−8.368, 4.959)	0.616	−1.627 (−8.254, 4.999)	0.63	−2.335 (−8.801, 4.132)	0.479

*^*a*^The model adjusted for age, sex, ethnicity, and education*

*^*b*^The model additionally adjusted for residence, marriage, exercise, smoking, and drinking alcohol based on the prior model*

*^*c*^The model additionally adjusted for BMI, hypertension, glucose for cholesterol outcome, TC for glucose outcome based on the prior model*

*Unit: mmol/L for TC, TG, HDLC, LDLC, and FBG, μmol/L for GSP.*

### The Association Between Lipids/Glucose and Cognitive Function

**FIGURE 2:**
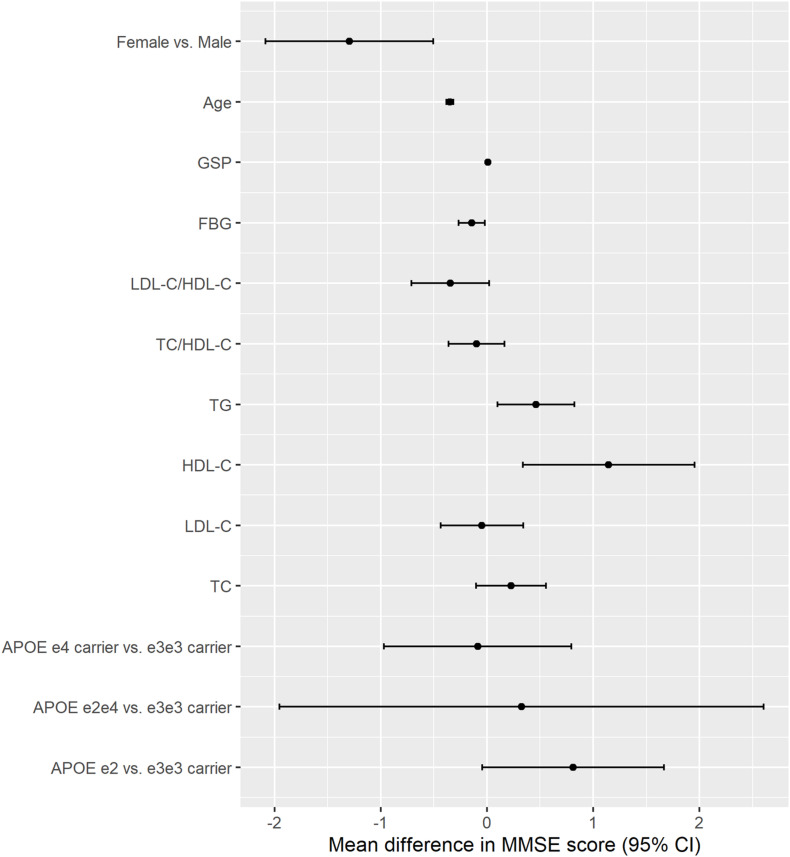
The association between *APOE*, lipids, glucose, age, sex and cognitive function (MMSE continuous score). The results were from models for *APOE*, age, sex, and each lipids and glucose biomarkers separately; All models adjusted for age, sex, ethnicity, education, residence, marriage, exercise, smoking, drinking alcohol, BMI, and hypertension; Unit: year for age, mmol/L for TC, TG, HDLC, LDLC, and FBG, μmol/L for GSP.

Higher TG, HDL-C, and GSP were associated with higher MMSE score [change in MMSE (95% CI): 0.461 (0.098, 0.825) for TG, 1.146 (0.336, 1.956) for HDL-C, 0.01 (0.003, 0.017) for GSP] (Figure 2 and Table 3). The increase of TC, TG, HDL-C, and GSP were associated with lower odds of cognitive dysfunction [OR (95% CI): 0.857 (0.775, 0.948) for TC, 0.836 (0.711, 0.983) for TG, 0.685 (0.519, 0.905) for HDL-C, 0.996 (0.993, 0.999) for GSP] (Supplementary Table 1). Higher LDL-C/HDL-C was only associated with lower MMSE score before adjusting for BMI, glucose and hypertension [change in MMSE (95% CI): −0.423 (−0.777, −0.069)] (Table 3). Besides, only those with FBG lower than 3.9 mmol/L had higher MMSE score and lower odds of cognitive dysfunction compared to those with normal FBG (3.9−6.1 mmol/L) [change in MMSE score (95% CI): 1.29 (0.674, 1.91), OR for cognitive dysfunction (95% CI): 0.779 (0.619, 0.980)]. There was no significant difference in cognitive function between participants with normal FBG and elevated FBG (≥6.1 mmol/L). Increasing age was associated with lower MMSE score [mean difference (95% CI): −0.35 (−0.383, −0.317)] and higher odds of cognitive dysfunction [OR (95% CI): 1.105 (1.093, 1.117)]. Female had lower MMSE score [mean difference (95% CI): −1.295 (−2.084, −0.505)] and higher odds of cognitive dysfunction compared to the male [OR (95% CI): 1.554 (1.193, 2.023)]. We found some significant interactions between age/sex and biomarkers on MMSE. The association between HDL-C and MMSE was stronger in the female than the male. The association between TC, HDL-C, and GSP and MMSE score all became stronger with the age increasing.

**TABLE 3:** The association between lipids, glucose and cognitive function (MMSE continuous score).

**Biomarker**	**Change in MMSE score (95% CI)^*a*^**	***p*-Value^*a*^**	**Change in MMSE score (95% CI)^*b*^**	***p*-Value^*b*^**	**Change in MMSE score (95% CI)^*c*^**	***p*-Value^*c*^**
Each mmol/L increment in TC	0.255 (−0.072, 0.581)	0.126	0.188 (−0.142, 0.518)	0.264	0.226 (−0.103, 0.555)	0.179
Each mmol/L increment in TG	0.415 (0.074, 0.755)	0.017	0.327 (−0.022, 0.676)	0.066	0.461 (0.098, 0.825)	0.013
Each mmol/L increment in HDL-C	1.167 (0.410, 1.925)	0.003	1.276 (0.501, 2.051)	0.001	1.146 (0.336, 1.956)	0.006
Each mmol/L increment in LDL-C	−0.018 (−0.400, 0.363)	0.925	−0.104 (−0.493, 0.286)	0.602	−0.047 (−0.436, 0.343)	0.815
TC/HDL-C	−0.108 (−0.342, 0.126)	0.367	−0.172 (−0.428, 0.084)	0.188	−0.098 (−0.362, 0.167)	0.469
LDL-C/HDL-C	−0.321 (−0.665, 0.023)	0.067	−0.423 (−0.777, −0.069)	0.019	−0.345 (−0.712, 0.021)	0.065
FBG	−0.136 (−0.264, −0.009)	0.036	−0.151 (−0.273, −0.029)	0.015	−0.144 (−0.266, −0.021)	0.022
GSP	0.011 (0.004, 0.017)	0.002	0.010 (0.003, 0.017)	0.004	0.010 (0.003, 0.017)	0.005

*^*a*^The models adjusted for age, sex, ethnicity, and education*

*^*b*^The models additionally adjusted for residence, marriage, exercise, smoking, and drinking alcohol based on the prior model*

*^*c*^The models additionally adjusted for BMI, hypertension, and glucose for cholesterol outcome, TC for glucose outcome based on the prior model.*

### The Association of *APOE*, Lipids, Glucose, and Cognitive Function

*APOE* ε2 carriers were associated with higher MMSE score and lower odds of cognitive dysfunction compared with *APOE* ε3ε3 carriers, and the difference increased after adjusting for TC or GSP [Mean difference in MMSE score (95% CI): 0.812 (−0.043, 1.668) vs. 0.908 (0.05, 1.765) adjusting for TC, 0.865 (0.012, 1.719) adjusting for GSP] (Table 4). *APOE*ε4 carriers had non-significant lower MMSE scores and higher odds of cognitive dysfunction than ε3ε3 carriers in our population. The mean differences of the MMSE score before and after adjusting for the TC were −0.086 (95% CI: −0.969, 0.796) and −0.113 (95% CI: −0.993, 0.766) between *APOE*ε4 carriers and ε3ε3 carriers (Table 4). *APOE* was not significantly associated with cognitive dysfunction in our study (Supplementary Table 2).

**TABLE 4:** The association among *APOE*, lipids, glucose and cognitive function (MMSE continuous score).

**Model**	**Variable**	**Change in MMSE score (95% CI)^*a*^**	***p*-Value^*a*^**	**Change in MMSE score (95% CI)^*b*^**	***p*-Value^*b*^**	**Change in MMSE score (95% CI)^*c*^**	***p*-Value^*c*^**
Model APOE	ε3ε3 carrier	Reference	/	Reference	/	Reference	/
	ε2 carrier	0.794 (−0.061, 1.648)	0.069	0.793 (−0.061, 1.647)	0.069	0.812 (−0.043, 1.668)	0.063
	ε2ε4 carrier	0.615 (−1.606, 2.835)	0.587	0.327 (−1.885, 2.539)	0.772	0.324 (−1.955, 2.603)	0.780
	ε4 carrier	−0.087 (−0.976, 0.801)	0.847	−0.098 (−0.975, 0.779)	0.827	−0.086 (−0.969, 0.796)	0.848
Model APOE + TC	ε3ε3 carrier	Reference	/	Reference	/	Reference	/
	ε2 carrier	0.916 (0.061, 1.771)	0.036	0.888 (0.032, 1.744)	0.042	0.908 (0.050, 1.765)	0.038
	ε2ε4 carrier	0.685 (−1.537, 2.907)	0.546	0.393 (−1.809, 2.595)	0.726	0.401 (−1.868, 2.669)	0.729
	ε4 carrier	−0.117 (−1.001, 0.768)	0.796	−0.121 (−0.994, 0.753)	0.787	−0.113 (−0.993, 0.766)	0.801
	Each mmol/L increment in TC	0.316 (−0.010, 0.642)	0.057	0.247 (−0.082, 0.577)	0.142	0.251 (−0.081, 0.583)	0.139
Model APOE + TG	ε3ε3 carrier	Reference	/	Reference	/	Reference	/
	ε2 carrier	0.771 (−0.085, 1.627)	0.077	0.779 (−0.075, 1.634)	0.074	0.797 (−0.059, 1.652)	0.068
	ε2ε4 carrier	0.544 (−1.657, 2.746)	0.628	0.290 (−1.914, 2.494)	0.797	0.298 (−1.972, 2.568)	0.797
	ε4 carrier	−0.106 (−0.992, 0.781)	0.815	−0.108 (−0.984, 0.767)	0.808	−0.103 (−0.984, 0.777)	0.818
	Each mmol/L increment in TG	0.401 (0.061, 0.742)	0.021	0.317 (−0.032, 0.666)	0.075	0.352 (−0.006, 0.709)	0.054
Model APOE + HDLC	ε3ε3 carrier	Reference	/	Reference	/	Reference	/
	ε2 carrier	0.753 (−0.098, 1.605)	0.083	0.751 (−0.102, 1.603)	0.084	0.768 (−0.086, 1.622)	0.078
	ε2ε4 carrier	0.467 (−1.735, 2.670)	0.677	0.246 (−1.899, 2.390)	0.822	0.222 (−1.982, 2.425)	0.844
	ε4 carrier	−0.04 (−0.928, 0.848)	0.93	−0.052 (−0.927, 0.823)	0.907	−0.037 (−0.917, 0.844)	0.935
	Each mmol/L increment in HDLC	1.129 (0.372, 1.886)	0.003	1.241 (0.467, 2.015)	0.002	1.199 (0.391, 2.007)	0.004
Model APOE + LDLC	ε3ε3 carrier	Reference	/	Reference	/	Reference	/
	ε2 carrier	0.826 (−0.033, 1.685)	0.059	0.785 (−0.073, 1.643)	0.073	0.810 (−0.051, 1.671)	0.065
	ε2ε4 carrier	0.646 (−1.583, 2.875)	0.570	0.320 (−1.893, 2.533)	0.777	0.322 (−1.959, 2.603)	0.782
	ε4 carrier	−0.096 (−0.984, 0.793)	0.833	−0.096 (−0.974, 0.782)	0.831	−0.086 (−0.970, 0.798)	0.849
	Each mmol/L increment in LDLC	0.072 (−0.312, 0.456)	0.712	−0.019 (−0.411, 0.372)	0.923	−0.006 (−0.402, 0.391)	0.978
Model APOE + TC/HDLC ratio	ε3ε3 carrier	Reference	/	Reference	/	Reference	/
	ε2 carrier	0.765 (−0.085, 1.615)	0.078	0.739 (−0.113, 1.590)	0.089	0.766 (−0.089, 1.621)	0.079
	ε2ε4 carrier	0.590 (−1.628, 2.808)	0.602	0.293 (−1.903, 2.489)	0.794	0.285 (−1.979, 2.549)	0.805
	ε4 carrier	−0.069 (−0.96, 0.823)	0.880	−0.064 (−0.944, 0.816)	0.887	−0.056 (−0.942, 0.831)	0.902
	TC/HDL-C	−0.072 (−0.301, 0.158)	0.541	−0.138 (−0.389, 0.113)	0.282	−0.115 (−0.381, 0.151)	0.397
Model APOE + LDL-C/HDLC ratio	ε3ε3 carrier	Reference	/	Reference	/	Reference	/
	ε2 carrier	0.687 (−0.163, 1.537)	0.113	0.648 (−0.203, 1.498)	0.136	0.672 (−0.183, 1.527)	0.123
	ε2ε4 carrier	0.505 (−1.696, 2.706)	0.653	0.212 (−1.958, 2.382)	0.848	0.192 (−2.041, 2.426)	0.866
	ε4 carrier	−0.047 (−0.939, 0.846)	0.918	−0.044 (−0.925, 0.838)	0.923	−0.029 (−0.916, 0.858)	0.949
	LDL-C/HDL-C	−0.261 (−0.604, 0.081)	0.135	−0.368 (−0.721, −0.015)	0.041	−0.349 (−0.724, 0.025)	0.067
Model APOE + FBG	ε3ε3 carrier	Reference	/	Reference	/	Reference	/
	ε2 carrier	0.757 (−0.098, 1.612)	0.083	0.758 (−0.097, 1.612)	0.082	0.777 (−0.079, 1.633)	0.075
	ε2ε4 carrier	0.591 (−1.618, 2.801)	0.600	0.334 (−1.866, 2.534)	0.766	0.323 (−1.938, 2.584)	0.780
	ε4 carrier	−0.097 (−0.982, 0.789)	0.830	−0.107 (−0.982, 0.767)	0.810	−0.094 (−0.974, 0.785)	0.834
	Each mmol/L increment in FBG	−0.13 (−0.258, −0.003)	0.045	−0.146 (−0.268, −0.023)	0.020	−0.138 (−0.261, −0.015)	0.028
Model APOE + GSP	ε3ε3 carrier	Reference	/	Reference	/	Reference	/
	ε2 carrier	0.845 (−0.007, 1.697)	0.052	0.846 (−0.007, 1.698)	0.052	0.865 (0.012, 1.719)	0.047
	ε2ε4 carrier	0.811 (−1.471, 3.093)	0.486	0.487 (−1.775, 2.749)	0.673	0.492 (−1.840, 2.825)	0.679
	ε4 carrier	−0.063 (−0.956, 0.830)	0.890	−0.077 (−0.957, 0.804)	0.864	−0.067 (−0.953, 0.818)	0.881
	Each μmol/L increment in GSP	0.011 (0.004, 0.017)	0.001	0.010 (0.003, 0.017)	0.003	0.010 (0.003, 0.017)	0.003

*^*a*^The models adjusted for age, sex, ethnicity, and education*

*^*b*^The models additionally adjusted for residence, marriage, exercise, smoking, and drinking alcohol based on the prior model*

*^*c*^The models additionally adjusted for BMI and hypertension based on the prior model.*

Using the mediation analysis framework, we calculated the natural direct and indirect effect of *APOE* on cognitive function to explore possible causality. *APOE*ε2 had significantly positive direct effect and negative indirect effect through TC on MMSE score [Natural direct effect (NDE): 1.119 (0.231, 2.007), Natural indirect effect (NIE): −0.174 (−0.336, −0.012)]. There were no significant indirect effects through TG, LDL-C, HDL-D, TC/HDL-C, LDL-C/HDL-C, FBG, or GSP. There was no significant indirect effect through cholesterol or glucose for *APOE* ε4 (Table 5).

**TABLE 5:** The direct and indirect effect through the lipids/glucose of *APOE* on cognitive function (MMSE continuous score).

Mediator	Effect decomposition*	ε2 carriers vs. non-ε2 carrier^†^		ε4 carriers vs. non-ε4 carrier^†^	
		Estimate (95% CI)	*p-*Value	Estimate (95% CI)	*p*-Value
TC	NDE	1.119 (0.231, 2.007)	0.014	−0.656 (−1.641, 0.330)	0.192
	NIE	−0.174 (−0.336, −0.012)	0.035	0.035 (−0.026, 0.097)	0.263
TG	NDE	0.925 (0.050, 1.799)	0.038	−0.517 (−1.472, 0.438)	0.288
	NIE	0.021 (−0.032, 0.074)	0.441	0.015 (−0.038, 0.070)	0.564
HDL-C	NDE	0.873 (−0.002, 1.748)	0.051	−0.357 (−1.342, 0.628)	0.477
	NIE	0.073 (−0.007, 0.152)	0.074	−0.070 (−0.154, 0.015)	0.105
LDL-C	NDE	1.008 (0.106, 1.909)	0.029	−0.678 (−1.708, 0.353)	0.197
	NIE	−0.062 (−0.277, 0.153)	0.571	−0.009 (−0.087, 0.069)	0.822
TC/HDL-C	NDE	0.917 (0.029, 1.804)	0.043	−0.611 (−1.631, 0.408)	0.240
	NIE	0.029 (−0.120, 0.178)	0.703	−0.050 (−0.152, 0.053)	0.341
LDL-C/HDL-C	NDE	0.817 (−0.079, 1.713)	0.074	−0.530 (−1.594, 0.534)	0.329
	NIE	0.129 (−0.067, 0.324)	0.197	−0.083 (−0.186, 0.021)	0.118
Glucose	NDE	1.079 (0.189, 1.969)	0.018	−0.484 (−1.440, 0.472)	0.321
	NIE	0.019 (−0.022, 0.060)	0.359	−0.007 (−0.041, 0.026)	0.675
GSP	NDE	1.099 (0.212, 1.987)	0.015	−0.525 (−1.488, 0.438)	0.285
	NIE	−0.001 (−0.070, 0.068)	0.979	−0.041 (−0.129, 0.047)	0.356

*NDE is natural direct effect, NIE is natural indirect effect

*^†^The model adjusted for age, sex, ethnicity, education, residence, marriage, exercise, smoking, drinking alcohol, BMI, hypertension, FBG (cholesterol model), and TC (glucose model)*

*The interaction term in *APOE2* model did not have a significant effect on the model results. *APOE2* model did not include the interaction term of *APOE* and cholesterol, while the *APOE4* model included the interaction term of *APOE* and cholesterol.*

There was a borderline significant interaction of *APOE* ε4 and LDL-C (coefficient estimate = −0.34, *P* = 0.055) on cognitive dysfunction (Supplementary Table 3). Compared with *APOE*ε4 non-carriers, the ε4 carriers were at a higher risk for cognitive dysfunction in participants with low LDL-C levels (OR: 1.773, 95%CI: 1.186, 2.648). The difference was not significant in participants with high LDL-C levels. *APOE* ε2 only showed a significant protective effect in participants with low FBG (OR for cognitive dysfunction: 0.582, 95%CI: 0.342, 0.992) (Supplementary Table 4).

In addition, we tested the association between *APOE* genotype and age, *APOE* genotype and sex on MMSE separately. We only found a significant interaction between *APOE* ε2 and sex, and *APOE* ε2 carriers had higher MMSE score than *APOE*ε3ε3 carriers only in the female, not in the male. We further found a significant three-way interaction of *APOE* ε2, sex and TC on cognitive dysfunction. The association between *APOE* ε2 carriers and cognitive dysfunction in the female increased with the increasing of TC.

## Discussion

We found the effect of *APOE* on cognitive function was mediated by cholesterol, but not by glucose. First, we found that *APOE* ε2 carriers (protective variant) had lower TC and LDL-C, and higher HDL-C compared to ε3ε3 carriers; ε4 carriers (risk variant) showed opposite associations. This supported the most recent evidence on our understanding of the role of APOE proteins and cholesterol. A meta-analysis in 2007 pooling 72,150 participants from 22 studies demonstrated an obvious increase trend of TC and LDL-C in the order of *APOE* ε2ε2, ε2ε3, ε3ε3, ε3ε4, and ε4ε4 (Bennet et al., 2007). In the Chinese population, studies also found *APOE* ε2 carriers had lower TC and LDL-C than *APOE* ε2 non-carriers (Kang et al., 2016), and *APOE* ε4 carriers had higher serum TC, TG, and LDL levels and lower HDL levels than *APOE* ε4 non-carriers (Wei et al., 2020). In addition, age was also negatively associated with TC. The difference of TC between *APOE* ε2 carriers and ε3ε3 carriers was equivalent to the increasing of 35 years in age (TC change: −0.418 vs. −0.012).

Second, when investigating the role of lipids, we found higher TC, TG, HDL-C, and LDL-C were all associated with lower risks of cognitive dysfunction among all participants. This association persisted in participants aged 75 and older but disappeared in participants aged from 65 to 74 (data not shown). However, not all prior evidence concurs with our findings, as a recent meta-analysis found that LDL-C cholesterol levels increase the risk for AD, whereas HDL-C, TC, and TG levels were not sensitive hallmarks of AD (Sáiz-Vazquez et al., 2020). Prior reviews have established an age-dependent association between cholesterol and cognitive function: high cholesterol levels were associated with an increased risk of cognitive decline in midlife (age 40–59) while showing no association with or decreased risk of cognitive function in late-life (age 60 and older) (Anstey et al., 2008; Van Vliet, 2012). Crichton et al. speculated that persons over 60 are survivors and thus less likely to show cognitive deficit with TC, LDL-C, and TG (Crichton et al., 2014). Another explanation was that cholesterol decreases with aging, which could be the result of underlying diseases like cognitive decline (Van Vliet, 2012).

We found a protective effect of *APOE* ε2 on cognitive function, which was mediated by TC and LDL-C in a suppressive way (Conger, 1974). The natural direct effect of *APOE* ε2 on MMSE score has the equivalent magnitude of about 3-year reduction in age (MMSE score difference of ε2 carriers vs. ε2 non-carriers: 1.119; each year increase of age: −0.350). *APOE* ε2 was a protective isoform for cognitive dysfunction, but it could also lower the TC and LDL-C level and led to a higher risk for cognitive dysfunction in late-life. One previous mediation study found the effect of *APOE* ε4 on cognition was mediated by cardiovascular biomarkers such as MRI markers in the same direction of the direct effect (Sajeev, 2016). However, we did not see a significant mediation effect from the cholesterol for the *APOE* ε4 but found a borderline significant interaction of *APOE* ε4 and LDL-C. *APOE* ε4 showed a harmful effect on cognitive function only in those with low LDL-C levels. This implied that the protective effect of high LDL-C in the elderly might counteract the harmful effect of *APOE* ε4. Another explanation could be the bias due to the lost follow-up of ε4 carriers due to CVD diseases in the high LDL-C group. Five among seven studies in a review detected no interaction between *APOE* ε4 and TC (Anstey et al., 2008), while some studies found the effect of cholesterol varied in *APOE* ε4 carriers and non-carriers (Hall et al., 2006; Wei et al., 2020). Possible explanations for the inconsistent results may be the differences in sample size, ethnicity, age, and other population characteristics. Our findings suggest that we need to consider the *APOE* polymorphism and cholesterol level simultaneously for the risk assessment and treatment of the cognitive dysfunction among the old Chinese population.

We found normal glucose group had lower MMSE score than the hypoglycemia group. But we did not found a significant difference between the abnormal high glucose group and the normal glucose group. In addition, we found *APOE* was not associated with FBG level and FBG may modify the effect of *APOE* ε2. These findings have been unable to demonstrate either the association between *APOE* and FBG or the positive association between diabetes and cognitive dysfunction found in previous studies (Shinohara and Sato, 2019). Prior studies also indicated coexistence of elevated blood glucose and *APOE* ε4 increased risk of cognitive dysfunction (Peila et al., 2002; Matsuzaki et al., 2010). *APOE* isoforms were found to differentially modulate brain insulin/insulin-like growth factor signaling and downstream glucose uptake and metabolism in the female brain (Keeney et al., 2015). It should be of note that only 7.4% of the people were considered to have diabetes in our study. The FBG level may also not be as sensitive as the glucose metabolism indicators in the brain for cognitive function.

Strengths of our study include the use of an elderly population cohort with an emphasis on the oldest old. The cohort has a comprehensive list of demographic characteristics, lifestyle, gene, and biomarkers information, covering a broad geographical area in China. The study population includes a higher proportion of the oldest-old population than other studies, with extended longitudinal follow-up for changes in cognitive function. Our study provides new quantitative evidence regarding the role of lipids and glucose in the association between *APOE* and cognitive function in an elderly population. Our study also has some limitations. First, we did not consider measures of changing lipids or glucose level over time, or information regarding drug treatment for dyslipidemia or dysglycemia for study participants. Failure to adjust for the medication could have led to confounding bias. Second, we did not have measures of brain lipids or brain glucose, which could more accurately reflect CNS and may be more biologically active than blood biomarkers. Third, the majority of our participants were elderly Han Chinese with little or no formal education due to the historical period of third childhood, and may have lasting social impacts on health. Lastly, this select group of individuals may have limited generalizability to elderly populations elsewhere in the world. Nevertheless, these findings are particularly pertinent in light of China’s demographic changes toward an increasingly elderly population. An understanding of gene-environment mediation and interaction among lipids, glucose and genetic pre-dispositions for cognitive dysfunction could inform prevention efforts.

## Conclusion

In conclusion, we found evidence suggesting that TC could mediate the effect of *APOE* ε2 on cognitive function negatively and that the effect of *APOE* ε4 could be modified by LDL-C level in an elderly Chinese population. We did not see abnormally high FBG increased the risk of cognitive dysfunction or mediated the effect of *APOE*, unlike prior research findings. These findings contribute to growing evidence of the genetic effects of *APOE* subtypes on cognitive function, and suggest the role of lipid and glucose metabolism in AD prevention. Further research is needed to characterize the causal pathways between *APOE* genotype, lipids, glucose and cognitive function and extend the generalizability of our findings.

## Data Availability Statement

The CLHLS datasets are publicly available at the Peking University Open Research Data on^1^. The dataset is publicly accessible to scholars for non-profit purposes.

## Ethics Statement

The studies involving human participants were reviewed and approved by the Research Ethics Committees of Duke University and Peking University (IRB00001052-13074). The patients/participants provided their written informed consent to participate in this study.

## Author Contributions

JJ and LL conceptualized the study, conducted statistical analysis, and drafted and edited the article. YZ acquired the data. HL, HI, and AL interpreted the results and revised the article. All authors provided critical insights and reviewed the article.

## Conflict of Interest

JJ received a research grant from Kunshan Government and travel support from ISEE in the past 36 months. The remaining authors authors declare that the research was conducted in the absence of any commercial or financial relationships that could be construed as a potential conflict of interest.

## Publisher’s Note

All claims expressed in this article are solely those of the authors and do not necessarily represent those of their affiliated organizations, or those of the publisher, the editors and the reviewers. Any product that may be evaluated in this article, or claim that may be made by its manufacturer, is not guaranteed or endorsed by the publisher.

## Footnotes

1
http://opendata.pku.edu.cn/dataverse/CHADS
