# Supplementary material for: Apolipoprotein E Induced Cognitive Dysfunction: Mediation Analysis of Lipids and Glucose Biomarkers in an Elderly Cohort Study
Source: Front Aging Neurosci. 2021 Aug 13;13:727289. doi: 10.3389/fnagi.2021.727289 (PMC8415114; doi:10.3389/fnagi.2021.727289)
Supplement: Supplementary file 1 [file Data_Sheet_1.docx]

# Supplementary materials

**Apolipoprotein E Induced Cognitive Dysfunction: Mediation Analysis of Lipids and Glucose Biomarkers in an Elderly Cohort Study**

Linxin **Liu** MMed, Huichu **Li** MA, Hari **Iyer** ScD, Andy J. **Liu**, MD, Yi **Zeng** PhD, John S. **Ji**^*^ ScD

**Table S1. The association between lipids/glucose and cognitive function (Binary cognitive dysfunction)**

**Table S2. The association between *APOE*, lipids, glucose and cognitive function (Binary cognitive dysfunction)**

**Table S3. The interaction of *APOE* and lipids/glucose on cognitive dysfunction**

**Table S4. The OR (95%CI) of the *APOE* for cognitive dysfunction stratified by different lipids/glucose level**

**Fig. S1. The inclusion of the study subjects**

**Table S1. The association between lipids/glucose and cognitive function (Binary cognitive dysfunction)**

| **Cognitive dysfunction (Yes vs. No)** | **OR (95% CI) ^a^** | **p value ^a^** |  | **OR (95% CI) ^b^** | **p value ^b^** |  | **OR (95% CI) ^c^** | **p value ^c^** |
| --- | --- | --- | --- | --- | --- | --- | --- | --- |
| Each mmol/L increment in TC | 0.851 (0.77, 0.941) | 0.002 |  | 0.865 (0.782, 0.956) | 0.005 |  | 0.857 (0.775, 0.948) | 0.003 |
| Each mmol/L increment in TG | 0.849 (0.724, 0.996) | 0.044 |  | 0.849 (0.724, 0.996) | 0.044 |  | 0.836 (0.711, 0.983) | 0.030 |
| Each mmol/L increment in HDL-C | 0.669 (0.514, 0.871) | 0.003 |  | 0.677 (0.518, 0.887) | 0.005 |  | 0.685 (0.519, 0.905) | 0.008 |
| Each mmol/L increment in LDL-C | 0.896 (0.793, 1.011) | 0.075 |  | 0.911 (0.806, 1.029) | 0.134 |  | 0.898 (0.795, 1.015) | 0.085 |
| TC/ HDL-C | 1.016 (0.941, 1.097) | 0.678 |  | 1.021 (0.948, 1.099) | 0.591 |  | 1.011 (0.934, 1.094) | 0.781 |
| LDL-C/ HDL-C | 1.032 (0.916, 1.163) | 0.603 |  | 1.043 (0.924, 1.178) | 0.496 |  | 1.028 (0.907, 1.166) | 0.664 |
| Each mmol/L increment in FBG | 1.016 (0.971, 1.064) | 0.487 |  | 1.018 (0.97, 1.067) | 0.468 |  | 1.02 (0.971, 1.072) | 0.429 |
| Each μmol/L increment in GSP | 0.996 (0.992, 0.999) | 0.011 |  | 0.996 (0.992, 0.999) | 0.011 |  | 0.996 (0.993, 0.999) | 0.035 |

Note: a. The models adjusted for age, sex, ethnicity, and education; b. The models additionally adjusted for residence, marriage, exercise, smoking, and drinking alcohol based on the prior model; c. The models additionally adjusted for BMI, hypertension, FBG (cholesterol model), TC (glucose model) based on the prior model.

**Table S2. The association between *APOE*, lipids, glucose and cognitive function (Binary cognitive dysfunction)**

| **Model** | **Variable** | **OR (95% CI)^a^** | **p value ^a^** |  | **OR (95% CI)^b^** | **p value ^b^** |  | **OR (95% CI)^c^** | **p value ^c^** |
| --- | --- | --- | --- | --- | --- | --- | --- | --- | --- |
| **Model APOE** | **ε3ε3 carrier** | Reference | / |  | Reference | / |  | Reference | / |
|  | **ε2 carrier** | 0.916 (0.693, 1.21) | 0.536 |  | 0.903 (0.682, 1.19) | 0.473 |  | 0.911 (0.689, 1.2) | 0.513 |
|  | **ε2ε4 carrier** | 1.232 (0.626, 2.42) | 0.546 |  | 1.396 (0.729, 2.68) | 0.314 |  | 1.36 (0.714, 2.59) | 0.35 |
|  | **ε4 carrier** | 1.213 (0.901, 1.63) | 0.203 |  | 1.211 (0.901, 1.63) | 0.204 |  | 1.232 (0.916, 1.66) | 0.168 |
| **Model APOE+TC** | **ε3ε3 carrier** | Reference | / |  | Reference | / |  | Reference | / |
|  | **ε2 carrier** | 0.848 (0.638, 1.127) | 0.256 |  | 0.842 (0.634, 1.12) | 0.238 |  | 0.841 (0.633, 1.118) | 0.234 |
|  | **ε2ε4 carrier** | 1.163 (0.583, 2.319) | 0.669 |  | 1.321 (0.685, 2.546) | 0.406 |  | 1.324 (0.678, 2.587) | 0.411 |
|  | **ε4 carrier** | 1.236 (0.922, 1.658) | 0.156 |  | 1.234 (0.921, 1.655) | 0.159 |  | 1.232 (0.918, 1.653) | 0.165 |
|  | **Each mmol/L increment in TC** | 0.837 (0.756, 0.926) | 0.001 |  | 0.85 (0.768, 0.941) | 0.002 |  | 0.85 (0.768, 0.941) | 0.002 |
| **Model APOE+TG** | **ε3ε3 carrier** | Reference | / |  | Reference | / |  | Reference | / |
|  | **ε2 carrier** | 0.923 (0.698, 1.222) | 0.577 |  | 0.911 (0.688, 1.206) | 0.514 |  | 0.908 (0.686, 1.203) | 0.502 |
|  | **ε2ε4 carrier** | 1.269 (0.662, 2.435) | 0.473 |  | 1.43 (0.755, 2.708) | 0.273 |  | 1.433 (0.743, 2.763) | 0.283 |
|  | **ε4 carrier** | 1.23 (0.916, 1.653) | 0.169 |  | 1.228 (0.914, 1.649) | 0.172 |  | 1.223 (0.91, 1.643) | 0.182 |
|  | **Each mmol/L increment in TG** | 0.844 (0.718, 0.991) | 0.038 |  | 0.845 (0.719, 0.992) | 0.039 |  | 0.844 (0.718, 0.993) | 0.041 |
| **Model APOE+HDLC** | **ε3ε3 carrier** | Reference | / |  | Reference | / |  | Reference | / |
|  | **ε2 carrier** | 0.928 (0.702, 1.226) | 0.599 |  | 0.912 (0.69, 1.207) | 0.521 |  | 0.911 (0.689, 1.206) | 0.516 |
|  | **ε2ε4 carrier** | 1.254 (0.621, 2.533) | 0.528 |  | 1.418 (0.733, 2.743) | 0.3 |  | 1.436 (0.739, 2.791) | 0.286 |
|  | **ε4 carrier** | 1.193 (0.886, 1.607) | 0.244 |  | 1.194 (0.888, 1.607) | 0.241 |  | 1.19 (0.885, 1.601) | 0.249 |
|  | **Each mmol/L increment in HDLC** | 0.676 (0.52, 0.88) | 0.004 |  | 0.685 (0.524, 0.895) | 0.006 |  | 0.685 (0.521, 0.9) | 0.007 |
| **Model APOE+LDLC** | **ε3ε3 carrier** | Reference | / |  | Reference | / |  | Reference | / |
|  | **ε2 carrier** | 0.858 (0.645, 1.141) | 0.293 |  | 0.853 (0.641, 1.137) | 0.278 |  | 0.851 (0.639, 1.132) | 0.268 |
|  | **ε2ε4 carrier** | 1.158 (0.581, 2.309) | 0.677 |  | 1.325 (0.686, 2.559) | 0.403 |  | 1.328 (0.677, 2.604) | 0.409 |
|  | **ε4 carrier** | 1.231 (0.917, 1.653) | 0.166 |  | 1.228 (0.915, 1.649) | 0.171 |  | 1.225 (0.912, 1.645) | 0.178 |
|  | **Each mmol/L increment in LDLC** | 0.873 (0.771, 0.99) | 0.034 |  | 0.889 (0.784, 1.007) | 0.065 |  | 0.886 (0.781, 1.005) | 0.06 |
| **Model APOE+TC/HDLC** | **ε3ε3 carrier** | Reference | / |  | Reference | / |  | Reference | / |
|  | **ε2 carrier** | 0.918 (0.695, 1.214) | 0.549 |  | 0.906 (0.685, 1.199) | 0.491 |  | 0.904 (0.683, 1.196) | 0.479 |
|  | **ε2ε4 carrier** | 1.234 (0.627, 2.429) | 0.543 |  | 1.401 (0.73, 2.686) | 0.310 |  | 1.411 (0.726, 2.741) | 0.31 |
|  | **ε4 carrier** | 1.21 (0.898, 1.631) | 0.210 |  | 1.208 (0.897, 1.627) | 0.214 |  | 1.203 (0.893, 1.62) | 0.225 |
|  | **TC/HDL-C** | 1.006 (0.931, 1.087) | 0.877 |  | 1.01 (0.937, 1.089) | 0.792 |  | 1.009 (0.933, 1.091) | 0.82 |
| **Model APOE+LDLC/HDLC** | **ε3ε3 carrier** | Reference | / |  | Reference | / |  | Reference | / |
|  | **ε2 carrier** | 0.923 (0.697, 1.222) | 0.575 |  | 0.913 (0.689, 1.211) | 0.528 |  | 0.91 (0.686, 1.206) | 0.512 |
|  | **ε2ε4 carrier** | 1.24 (0.63, 2.44) | 0.533 |  | 1.411 (0.736, 2.705) | 0.300 |  | 1.421 (0.732, 2.759) | 0.3 |
|  | **ε4 carrier** | 1.209 (0.898, 1.629) | 0.212 |  | 1.206 (0.896, 1.624) | 0.217 |  | 1.201 (0.892, 1.617) | 0.228 |
|  | **LDL-C/HDL-C** | 1.017 (0.901, 1.149) | 0.781 |  | 1.028 (0.908, 1.164) | 0.663 |  | 1.025 (0.902, 1.165) | 0.706 |
| **Model APOE+FBG** | **ε3ε3 carrier** | Reference | / |  | Reference | / |  | Reference | / |
|  | **ε2 carrier** | 0.918 (0.694, 1.212) | 0.545 |  | 0.904 (0.683, 1.196) | 0.479 |  | 0.902 (0.682, 1.193) | 0.469 |
|  | **ε2ε4 carrier** | 1.234 (0.629, 2.422) | 0.541 |  | 1.397 (0.73, 2.673) | 0.313 |  | 1.407 (0.726, 2.728) | 0.312 |
|  | **ε4 carrier** | 1.213 (0.902, 1.631) | 0.202 |  | 1.211 (0.901, 1.628) | 0.205 |  | 1.206 (0.897, 1.621) | 0.215 |
|  | **Each mmol/L increment in FBG** | 1.016 (0.97, 1.064) | 0.499 |  | 1.017 (0.97, 1.067) | 0.483 |  | 1.016 (0.967, 1.067) | 0.53 |
| **Model APOE+GSP** | **ε3ε3 carrier** | Reference | / |  | Reference | / |  | Reference | / |
|  | **ε2 carrier** | 0.906 (0.688, 1.194) | 0.485 |  | 0.892 (0.676, 1.178) | 0.422 |  | 0.89 (0.674, 1.175) | 0.41 |
|  | **ε2ε4 carrier** | 1.143 (0.556, 2.35) | 0.717 |  | 1.296 (0.653, 2.573) | 0.459 |  | 1.301 (0.646, 2.622) | 0.461 |
|  | **ε4 carrier** | 1.21 (0.896, 1.632) | 0.213 |  | 1.209 (0.897, 1.629) | 0.213 |  | 1.202 (0.892, 1.621) | 0.226 |
|  | **Each μmol/L increment in GSP** | 0.996 (0.992, 0.999) | 0.011 |  | 0.996 (0.992, 0.999) | 0.011 |  | 0.996 (0.992, 0.999) | 0.011 |

Note: a. The model adjusted for age, sex, ethnicity, and education; b. The model additionally adjusted for residence, marriage, exercise, smoking, and drinking alcohol based on the prior model; c. The model additionally adjusted for BMI and hypertension based on the prior model.

**Table S3. The interaction of *APOE* and lipids/glucose on cognitive dysfunction**

| Variable | MMSE score | | |  | Cognitive dysfunction | | |
| --- | --- | --- | --- | --- | --- | --- | --- |
|  | coefficient | standard error | p value |  | coefficient | standard error | p value |
| ε3ε3 carrier | / | / | / |  | / | / | / |
| ε2 carrier | -0.216 | 1.993 | 0.914 |  | 0.066 | 0.613 | 0.914 |
| ε2ε4 carrier | -4.39 | 6.294 | 0.485 |  | 3.261 | 2.547 | 0.2 |
| ε4 carrier | -3.276 | 2.259 | 0.147 |  | 1.243 | 0.655 | 0.058 |
| TC | 0.115 | 0.194 | 0.552 |  | -0.119 | 0.06 | 0.048 |
| TC*ε2 carrier | 0.267 | 0.453 | 0.556 |  | -0.056 | 0.147 | 0.703 |
| TC*ε2ε4 | 1.173 | 1.47 | 0.425 |  | -0.738 | 0.634 | 0.245 |
| TC*ε4 carrier | 0.704 | 0.489 | 0.15 |  | -0.233 | 0.146 | 0.11 |
| ε3ε3 carrier | / | / | / |  | / | / | / |
| ε2 carrier | 0.644 | 0.825 | 0.435 |  | -0.049 | 0.259 | 0.848 |
| ε2ε4 carrier | 0.2 | 1.976 | 0.919 |  | 1.073 | 0.701 | 0.126 |
| ε4 carrier | -0.718 | 0.769 | 0.35 |  | 0.453 | 0.301 | 0.132 |
| TG | 0.26 | 0.2 | 0.195 |  | -0.117 | 0.096 | 0.221 |
| TG*ε2 carrier | 0.151 | 0.535 | 0.778 |  | -0.05 | 0.196 | 0.799 |
| TG*ε2ε4 | 0.094 | 1.689 | 0.956 |  | -0.718 | 0.651 | 0.27 |
| TG*ε4 carrier | 0.598 | 0.579 | 0.302 |  | -0.254 | 0.262 | 0.333 |
| ε3ε3 carrier | / | / | / |  | / | / | / |
| ε2 carrier | 0.605 | 1.463 | 0.679 |  | 0.009 | 0.542 | 0.987 |
| ε2ε4 carrier | -2.282 | 3.211 | 0.477 |  | 0.766 | 1.12 | 0.494 |
| ε4 carrier | -0.304 | 1.616 | 0.851 |  | -0.006 | 0.493 | 0.991 |
| HDLC | 1.112 | 0.491 | 0.023 |  | -0.381 | 0.167 | 0.022 |
| HDLC*ε2 carrier | 0.124 | 1.068 | 0.907 |  | -0.077 | 0.4 | 0.847 |
| HDLC*ε2ε4 | 1.778 | 1.724 | 0.303 |  | -0.3 | 0.759 | 0.692 |
| HDLC*ε4 carrier | 0.209 | 1.219 | 0.864 |  | 0.143 | 0.371 | 0.7 |
| ε3ε3 carrier | / | / | / |  | / | / | / |
| ε2 carrier | 0.197 | 1.363 | 0.885 |  | -0.063 | 0.436 | 0.885 |
| ε2ε4 carrier | 6.801 | 6.35 | 0.284 |  | -0.816 | 1.984 | 0.681 |
| ε4 carrier | -2.486 | 1.668 | 0.136 |  | 1.129 | 0.493 | 0.022 |
| LDLC | -0.147 | 0.231 | 0.525 |  | -0.07 | 0.074 | 0.346 |
| LDLC*ε2 carrier | 0.25 | 0.534 | 0.639 |  | -0.034 | 0.184 | 0.854 |
| LDLC*ε2ε4 | -3.089 | 2.871 | 0.282 |  | 0.515 | 0.856 | 0.547 |
| LDLC*ε4 carrier | 0.869 | 0.576 | 0.131 |  | -0.34 | 0.177 | 0.055 |
| ε3ε3 carrier | / | / | / |  | / | / | / |
| ε2 carrier | 0.879 | 1.388 | 0.526 |  | -0.16 | 0.449 | 0.721 |
| ε2ε4 carrier | 5.425 | 3.083 | 0.078 |  | 0.19 | 1.314 | 0.885 |
| ε4 carrier | -1.661 | 0.951 | 0.081 |  | 0.455 | 0.438 | 0.299 |
| TC/HDL-C ratio | -0.219 | 0.163 | 0.18 |  | 0.028 | 0.06 | 0.647 |
| TC/HDL-C*ε2 carrier | -0.049 | 0.371 | 0.895 |  | 0.021 | 0.128 | 0.867 |
| TC/HDL-C*ε2ε4 | -1.644 | 1.137 | 0.148 |  | 0.05 | 0.406 | 0.901 |
| TC/HDL-C*ε4 carrier | 0.423 | 0.214 | 0.048 |  | -0.072 | 0.111 | 0.515 |
| ε3ε3 carrier | / | / | / |  | / | / | / |
| ε2 carrier | 0.678 | 1.082 | 0.531 |  | -0.137 | 0.357 | 0.701 |
| ε2ε4 carrier | 5.559 | 1.475 | <0.001 |  | -0.36 | 0.909 | 0.692 |
| ε4 carrier | -1.619 | 1.323 | 0.221 |  | 0.819 | 0.434 | 0.059 |
| LDL-C/HDL-C ratio | -0.416 | 0.218 | 0.057 |  | 0.055 | 0.077 | 0.474 |
| LDL-C/HDL-C*ε2 carrier | -0.019 | 0.489 | 0.969 |  | 0.033 | 0.175 | 0.852 |
| LDL-C/HDL-C*ε2ε4 | -3.185 | 0.963 | 0.001 |  | 0.415 | 0.462 | 0.369 |
| LDL-C/HDL-C*ε4 carrier | 0.681 | 0.526 | 0.196 |  | -0.276 | 0.18 | 0.126 |
| ε3ε3 carrier | / | / | / |  | / | / | / |
| ε2 carrier | 1.117 | 1.273 | 0.38 |  | -0.185 | 0.409 | 0.652 |
| ε2ε4 carrier | 0.488 | 2.57 | 0.849 |  | -0.071 | 1.371 | 0.959 |
| ε4 carrier | 0.524 | 1.116 | 0.639 |  | 0.256 | 0.318 | 0.42 |
| FBG | -0.115 | 0.065 | 0.076 |  | 0.016 | 0.03 | 0.598 |
| FBG*ε2 carrier | -0.075 | 0.281 | 0.788 |  | 0.018 | 0.084 | 0.832 |
| FBG*ε2ε4 | -0.034 | 0.5 | 0.945 |  | 0.092 | 0.289 | 0.751 |
| FBG*ε4 carrier | -0.134 | 0.235 | 0.568 |  | -0.015 | 0.057 | 0.793 |
| ε3ε3 carrier | / | / | / |  | / | / | / |
| ε2 carrier | -0.944 | 3.543 | 0.79 |  | 0.651 | 1.102 | 0.555 |
| ε2ε4 carrier | 14.034 | 8.137 | 0.085 |  | -5.374 | 3.128 | 0.086 |
| ε4 carrier | 2.872 | 2.69 | 0.286 |  | -0.824 | 0.851 | 0.333 |
| GSP | 0.011 | 0.004 | 0.006 |  | -0.005 | 0.002 | 0.032 |
| GSP*ε2 carrier | 0.008 | 0.015 | 0.599 |  | -0.003 | 0.005 | 0.476 |
| GSP*ε2ε4 | -0.061 | 0.039 | 0.115 |  | 0.025 | 0.014 | 0.078 |
| GSP*ε4 carrier | -0.012 | 0.011 | 0.269 |  | 0.004 | 0.004 | 0.229 |

Note: All models adjusted for age, sex, ethnicity, education, residence, marriage, exercise, smoking, drinking alcohol, BMI and hypertension. Unit: mmol/L for TC, TG, HDLC, and LDLC.

**Table S4. The OR (95%CI) of the *APOE* for cognitive dysfunction stratified by different lipids/glucose level**

| **Biomarker group** | **Genotype** | **n** | **OR (95% CI)** | **p value** |
| --- | --- | --- | --- | --- |
| TC <3.8 mmol/L | **ε4 carriers vs. ε4 non-carriers** | 115 vs. 902 | 1.533 (0.901, 2.608) | 0.115 |
|  | **ε2 carriers vs. ε2 non-carriers** | 248 vs. 769 | 0.848 (0.552, 1.303) | 0.453 |
| TC ≥ 3.8 mmol/L | **ε4 carriers vs. ε4 non-carriers** | 330 vs. 1997 | 1.138 (0.797, 1.624) | 0.477 |
|  | **ε2 carriers vs. ε2 non-carriers** | 313 vs. 2014 | 0.837 (0.584, 1.2) | 0.333 |
| TG <1.7 mmol/L | **ε4 carriers vs. ε4 non-carriers** | 403 vs. 2611 | 1.291 (0.951, 1.751) | 0.102 |
|  | **ε2 carriers vs. ε2 non-carriers** | 501 vs. 2513 | 0.862 (0.645, 1.153) | 0.317 |
| TG ≥1.7 mmol/L | **ε4 carriers vs. ε4 non-carriers** | 42 vs. 288 | 0.93 (0.328, 2.642) | 0.892 |
|  | **ε2 carriers vs. ε2 non-carriers** | 60 vs. 270 | 0.693 (0.312, 1.54) | 0.368 |
| HDLC < 1 mmol/L | **ε4 carriers vs. ε4 non-carriers** | 102 vs. 594 | 1.445 (0.843, 2.475) | 0.181 |
|  | **ε2 carriers vs. ε2 non-carriers** | 100 vs. 596 | 0.733 (0.378, 1.42) | 0.357 |
| HDLC ≥1 mmol/L | **ε4 carriers vs. ε4 non-carriers** | 343 vs. 2305 | 1.178 (0.837, 1.659) | 0.347 |
|  | **ε2 carriers vs. ε2 non-carriers** | 461 vs. 2187 | 0.956 (0.706, 1.294) | 0.771 |
| LDLC <2.6 mmol/L | **ε4 carriers vs. ε4 non-carriers** | 196 vs. 1606 | 1.773 (1.186, 2.648) | **0.005** |
|  | **ε2 carriers vs. ε2 non-carriers** | 418 vs. 1384 | 0.809 (0.584, 1.12) | 0.202 |
| LDLC ≥2.6 mmol/L | **ε4 carriers vs. ε4 non-carriers** | 249 vs. 1293 | 0.885 (0.581, 1.349) | 0.57 |
|  | **ε2 carriers vs. ε2 non-carriers** | 143 vs. 1399 | 0.95 (0.52, 1.734) | 0.868 |
| FBG <3.9 mmol/L | **ε4 carriers vs. ε4 non-carriers** | 161 vs. 890 | 1.269 (0.76, 2.119) | 0.363 |
|  | **ε2 carriers vs. ε2 non-carriers** | 180 vs. 871 | 0.582 (0.342, 0.992) | **0.047** |
| 3.9≤ FBG <6.1 mmol/L | **ε4 carriers vs. ε4 non-carriers** | 225 vs. 1699 | 1.303 (0.871, 1.95) | 0.197 |
|  | **ε2 carriers vs. ε2 non-carriers** | 327 vs. 1597 | 1.025 (0.711, 1.478) | 0.895 |
| FBG ≥6.1 mmol/L | **ε4 carriers vs. ε4 non-carriers** | 59 vs. 310 | 0.898 (0.429, 1.879) | 0.775 |
|  | **ε2 carriers vs. ε2 non-carriers** | 54 vs. 315 | 0.99 (0.448, 2.185) | 0.979 |

Note: All models adjusted for age, sex, ethnicity, education, residence, marriage, exercise, smoking, drinking alcohol, BMI, and hypertension.

**
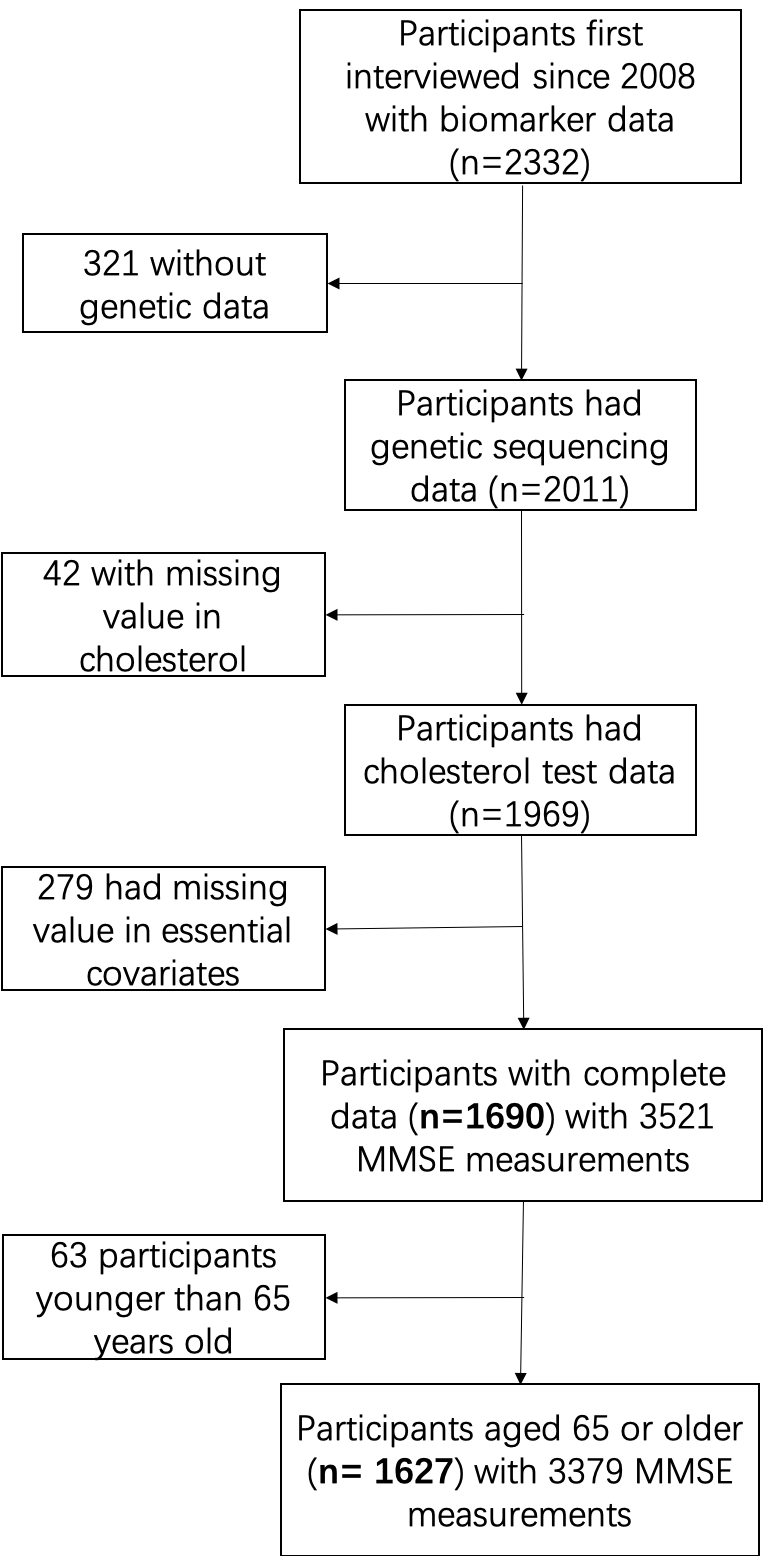
**

**Fig. S1. The inclusion of the study subjects**
